# Investigating Left Atrial Diameter and Heart Failure Onset in Middle‐Aged and Elderly: A Retrospective‐Prospective Study

**DOI:** 10.1002/clc.70085

**Published:** 2025-03-07

**Authors:** Ai Wang, Huilin Hu, Dai Zhang, Gang Qian, Haihua Pan, Changlin Zhai, Yan Yan

**Affiliations:** ^1^ Department of Cardiovascular Medicine, Zhongshan Hospital Fudan University Shanghai China; ^2^ Department of Cardiovascular Diseases The Affiliated Hospital of Jiaxing University Jiaxing China; ^3^ Key Laboratory for Carcinogenesis and Cancer Invasion (Ministry of Education), Department of Hepatic Oncology, Liver Cancer Institute, Zhongshan Hospital Fudan University Shanghai China

**Keywords:** echocardiography, heart failure, heart remodeling, retrospective‐prospective study

## Abstract

**Background:**

Left atrium (LA) is an integral component of left heart remodeling, reflecting hemodynamics and ventricular status. It remains uncertain whether left atrial diameter (LAD) can be utilized for predicting and evaluating the occurrence of heart failure (HF) in middle‐aged and elderly individuals.

**Methods:**

The study aimed to explore the correlation between LAD and HF in middle‐aged and elderly individuals, elucidating the timing of occurrence HF in relation to LAD. The retrospective‐prospective study investigated 4025 patients who underwent echocardiography at Zhongshan Hospital's Cardiovascular Department from January 2015 to December 2017. Patients were continuously monitored for HF until January 31, 2024. Cox regression analyses related baseline LAD to HF incidence, adjusted for known risk factors.

**Results:**

A total of 4025 individuals (mean age: 55 years, 45.29% male) were studied, spanning ages 45–91. Fifty‐one developed HF during a median follow‐up of 4.36 years. Cox regression model demonstrated the association between HF and LAD (HR = 5.721, 95% CI 3.768–8.687, *p* < 0.001) even after adjusting for covariates (age, weight, eGFR, HDL‐C, lymphocyte count, systolic blood pressure, FPG, HbA1C, waist circumference, hip circumference, valvular disease history, atrial fibrillation history).

**Conclusions:**

The link between LAD and future HF occurrence risk among middle‐aged and older adults shows a dose–response pattern. This relationship persists post‐adjustment for HF‐related factors, highlighting the predictive value of LAD in forecasting HF incidence.

## Graphical abstract

The association between LAD and future HF risk among middle‐aged and older adults shows a dose–response pattern. This relationship persists after adjusting for HF‐related factors, highlighting the predictive value of LAD in forecasting HF incidence.

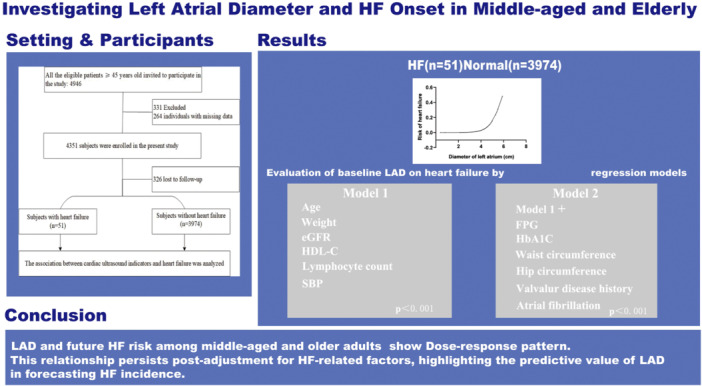

## 1 Introduction

Heart failure (HF) represents a serious manifestation or late‐stage complication of various cardiovascular diseases, affecting 1%–3% of adults worldwide [1, 2]. The incidence of HF increases with age, affecting more than 4% of people aged 65–70 [3, 4, 5]. Combined with a higher likelihood of developing other comorbidities and experiencing disability, this contributes to worse prognoses for the elderly [6].

HF, as a chronic disease, exhibits varying degrees of severity across different stages, each with distinct prognosis and varying probabilities of complications [7]. It is undeniable that intervening earlier in the course of impending HF can reduce its occurrence and associated complications. Especially in the elderly, those with HF have more cardiovascular events and adverse outcomes than those without HF. Therefore, identifying senior citizens at high risk of HF to initiate preventive measures before its initial onset is crucial for preventing HF and halting its progression.

Compared to blood markers such as B‐type natriuretic peptide, which are limited in their time‐consuming and usability, especially in emergency care settings, echocardiography is the most appropriate method to achieve these purposes due to its wide availability, safety, adaptability, and ability to provide real‐time imaging with excellent temporal and spatial resolution. In many current HF prediction models, the indicator of cardiac ultrasound is used, and this indicator is mostly left ventricular ejection fraction. Recent research has increasingly focused on the role of atrial remodeling in HF development. In addition to predicting atrial fibrillation, stroke, and mortality, left atrial enlargement and dysfunction can serve as markers of diastolic and systolic dysfunction [8, 9, 10, 11, 12, 13]. It has been reported that left atrial volume is a morphophysiological indicator of diastolic dysfunction [14, 15]. In patients with HF, left atrial size provides more predictive information than left ventricular function [16, 17]. An increased left atrial volume and dimensions indicate a higher risk of cardiac events for individuals with HF, regardless of their left ventricular ejection fraction [18]. It has been suggested that LAD and HF may be associated independently [19], although further research is required to confirm this finding. However, there is currently a lack of studies examining the predictive value of LAD in echocardiography for HF in individuals without pre‐existing HF, a stage with significant research potential.

Our research aims to conduct a retrospective‐prospective study, analyzing the relationship between left atrial size and the risk of HF in middle‐aged and elderly patients. In contrast, our study focuses on exploring the factors associated with the occurrence of HF among a diverse group of hospitalized individuals without pre‐existing HF. Our study was designed with the following objectives in mind to investigate this clinical hypothesis: (1) To study the morphological, functional, and remodeling characteristics of LA of elderly inpatients treated by the Cardiovascular Department. (2) To investigate the usefulness of echocardiographic LAD, especially as a predictive tool for HF.

## 2 Methods

### 2.1 Participants

This retrospective‐prospective study includes 4025 inpatients aged 45–91 years (minimum 45, maximum 91, median 55) from the Cardiology Department at Zhongshan Hospital, Fudan University, between 2015 and 2017. Follow‐up is up to January 31, 2024. All participants signed an informed consent form. The study protocol received a priori approval from the human research committee at Zhongshan Hospital, ensuring compliance with the ethical standards of the 1975 Declaration of Helsinki. The time of diagnosis of HF and the admission time due to HF were also documented for the patients.

### 2.2 Eligibility Criteria

The inclusion criteria for this study involve individuals aged over 45 who are admitted to the Cardiovascular Department, and it is open to both males and females. Conversely, the exclusion criteria encompass individuals with malignant tumors, pregnant individuals, those diagnosed with mental disorders, and those with a history of heart transplantation.

### 2.3 Documents and Measures

We included all patients with comprehensive information, covering age, sex, race, height, weight, education level, income, smoking habits, alcohol history, blood pressure, heart rate, serum creatinine, blood glucose level, LDL‐C, HDL‐C, triglycerides, cholesterol, white blood cell count, lymphocyte count, waist‐to‐hip ratio, as well as routine serial and transthoracic echocardiograms, along with medical history. The echocardiographic recordings included measurements of key parameters such as LAD, interventricular septal thickness at end‐diastole (IVSd), left ventricular internal dimension at end‐diastole (LVIDd), left ventricular internal dimension at end‐systole (LVIDs), posterior wall thickness at end‐diastole (PWTd), relative wall thickness, early diastolic mitral inflow velocity, late diastolic mitral inflow velocity, and the ratio of early to late diastolic mitral inflow velocities. Additionally, E‐wave deceleration time, left ventricular end‐diastolic volume, stroke volume, left ventricular ejection fraction, fractional shortening, aortic valve, tissue Doppler imaging, and mitral annular early diastolic velocity were also recorded. The following indices were calculated using the specified formulas: LVMI = [(0.8 × {1.04 [(LVIDd + PWTd + IVSd)^3^ − LVIDd^3^]} + 0.6)/height^2.7^].

LAD was measured using a standard 2D transthoracic echocardiogram with a (Philips Healthcare, Netherlands). The LAD measurements were obtained in the parasternal long‐axis view, at the end of ventricular systole, when the LA is at its largest dimension. The diameter was measured at the widest point of the LA from the inner edge to the inner edge of the opposite atrial wall. To ensure reliability, LAD was measured three times during each examination by trained echocardiographers, and the average value was used for analysis. All measurements were recorded in millimeters, and the results were reviewed for consistency.

### 2.4 Follow‐Up and Endpoint

Patients were followed up, evidence of HF was collected (subject to the diagnosis certificate of a tertiary hospital), and the time of HF was determined. All participants were subjected to a statistical analysis regarding the occurrence of HF, with each event adjudicated by a panel of three physicians. Follow‐up endpoint involved patients being diagnosed with HF, withdrawing from the study for various reasons, experiencing mortality, or not developing HF as of January 31, 2024.

### 2.5 Statistical Analysis

The formula for calculating body mass index (BMI) was mean weight divided by mean height squared (kg/m^2^). Systolic blood pressure (SBP) of at least 140 mmHg and/or diastolic blood pressure (DBP) of at least 90 mmHg were considered hypertension. Subjects receiving plasma glucose‐lowering treatment at baseline were also classified as diabetics. Diabetes was diagnosed based on fasting plasma glucose (FPG) values of 7.0 mmol/L and/or a documented history of prior diabetes. R studio or SPSS version 25.0 were used for all statistical analyses. Categorical data were shown as numbers and percentages, and continuous variables were reported as mean values ± standard deviations. Each increase of 1 standard deviation in LAD measurements was assessed for hazard ratios (HR). A Cox regression model, adjusted for potential confounders such as age, weight, eGFR, HDL‐C, lymphocyte count, SBP, FPG, HbA1c, waist circumference, hip circumference, history of valvular disease, and history of MI, was used to determine the independent association between LAD and the risk of HF, along with 95% confidence intervals (CIs). Subgroup analyses were conducted to explore how LAD relates to HF risk across age (< 55 and ≥ 55 years), sex, smoking, drinking, SBP (< 140 and ≥ 140 mmHg), FPG (< 7.0 and ≥ 7.0 mmol/L), and BMI (< 28 and ≥ 28 kg/m^2^) categories. Interaction tests within the Cox regression model were used to examine HR differences among these subgroups.

## 3 Results

### 3.1 Flow Chart

**Figure 1:**
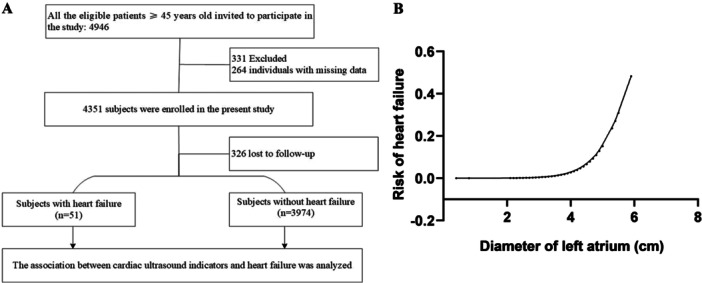
(A) Flowchart depicting the selection process and derivation of the study population. (B) Association between left atrial diameter and risk of heart failure after adjustment for various covariates.

A total of 4946 individuals underwent a review of medical history and clinical examination data. Eventually, 4351 individuals participated in the follow‐up, resulting in a successful final follow‐up of 4025 individuals. Among them, 51 cases of HF were identified. The median follow‐up time was 4.36 years. Detailed information can be found in the flowchart presented in Figure 1A.

### 3.2 Echocardiographic Characteristics of Study Participants

The study population consisted of 4025 patients, among whom 51 (1.27%) developed HF. Subjects with pre‐existing HF at baseline were excluded from the HF group. All the individuals were included in the analysis of baseline atrial diameters. Table 1 presents the baseline echocardiographic characteristics of the entire population, categorized based on the occurrence of HF during the follow‐up period.

**Table 1:** Baseline echocardiographic characteristics of the study population stratified by heart failure onset during follow‐up.

	HF (*n* = 51)	Normal (*n* = 3974)	*p*
AOD, cm	2.28 ± 0.22	2.24 ± 0.25	0.30
LAD, cm	3.79 ± 0.63	3.38 ± 0.40	< 0.001
IVSd. cm	0.91 ± 0.13	0.90 ± 0.25	0.82
LVIDd, cm	5.24 ± 0.64	4.71 ± 0.46	< 0.001
LVIDs, cm	3.62 ± 0.63	3.06 ± 0.34	< 0.001
PWTd, cm	0.89 ± 0.12	0.88 ± 0.34	0.94
LVMI, g/m^2^	45.68 ± 11.92	39.34 ± 37.96	0.24
RWT	0.34 ± 0.06	0.39 ± 0.33	0.37
E wave, cm/s	69.30 ± 24.47	71.46 ± 19.73	0.53
A wave, cm/s	83.33 ± 22.59	75.55 ± 24.32	0.03
E/A	0.89 ± 0.57	1.00 ± 0.48	0.12
EDT, ms	183.30 ± 37.74	191.84 ± 36.06	0.1
LVEDV, mL	132.76 ± 36.69	105.74 ± 29.39	< 0.001
SV, mL	75.70 ± 14.64	67.54 ± 19.19	0.003
LVEF, %	58.50 ± 7.80	63.91 ± 4.31	< 0.001
FS, %	31.11 ± 5.68	34.52 ± 11.60	0.038
AV	124.56 ± 42.70	120.43 ± 21.09	0.50
TDI	9.89 ± 13.73	8.69 ± 4.14	0.64
a′	12.26 ± 12.86	10.53 ± 3.87	0.48

Abbreviations: AOD, aortic root diameter; LAD, left atrial dimension; IVSd, interventricular septal thickness at end‐diastole; LVIDd, left ventricular internal dimension at end‐diastole; LVIDs, left ventricular internal dimension at end‐systole; PWTd, posterior wall thickness at end‐diastole; LVMI, left ventricular mass index; RWT, relative wall thickness; E wave, early diastolic mitral inflow velocity; A wave, late diastolic mitral inflow velocity; E/A, ratio of early to late diastolic mitral inflow velocities; EDT, E‐wave deceleration time; LVEDV, left ventricular end‐diastolic volume; SV, stroke volume; LVEF, left ventricular ejection fraction; FS, fractional shortening; AV, aortic valve; TDI, tissue Doppler imaging; a′, mitral annular early diastolic velocity.

### 3.3 Characteristics of Subjects Stratified by HF

The population is further categorized based on the presence or absence of HF. Table 2 presents the clinical and demographic characteristics of the study population, with a HF prevalence rate of 56.86%. In terms of demographic data, there were no significant differences observed between HF patients and the non‐HF group regarding age, history of smoking or alcohol consumption, educational level, and annual family income. Additionally, HF subjects were less likely to be currently smoking or consuming alcohol. Regarding anthropometric characteristics, the HF group showed significant increases in weight, with no changes observed in height and BMI. Consistent with this, adults with HF exhibited higher SBP levels and decreased eGFR, along with significant increases in waist circumference, hip circumference, and WHR. As expected, a higher proportion of participants with HF had a history of valve disease, atrial fibrillation, hypertension, and diabetes compared to those without HF. In terms of lipid profile, HF patients showed no significant differences in total cholesterol, triglycerides, and LDL‐C compared to the control group, while HDL‐C was significantly higher in the HF group (all *p* < 0.05). Finally, HF patients exhibited significant increases in lymphocyte count and blood glucose levels.

**Table 2:** Characteristics of subjects stratified by heart failure.

Variables	Heart failure (*N* = 51)	Non‐heart failure (*N* = 3974)	*p* value^a^
Age (years)	60.69 ± 10.13	55.39 ± 10.66	**< 0.001**
Male (%)	29 (56.86)	1794 (45.14)	0.095
Race (Han) (%)	49 (96.08)	3906 (98.29)	0.715
Height (cm)	162.53 ± 7.84	160.67 ± 8.24	0.109
Weight (kg)	66.81 ± 12.09	62.75 ± 11.50	**0.012**
BMI (kg/m^2^)	25.22 ± 3.73	24.25 ± 3.74	0.064
Education level (%)			0.067
Primary school or below	36 (70.59)	2192 (55.16)	
Junior and senior high school	14 (27.45)	1718 (43.23)	
Higher education	1 (1.96)	64 (1.61)	
Income (CNY) (%)	2.51 ± 0.70	2.42 ± 0.59	0.083
≤ 5000	11 (21.57)	723 (18.19)	
5000–20 000	33 (64.71)	2147 (54.03)	
＞20 000	7 (13.73)	1104 (27.78)	
Smoking history (%)	16 (31.37)	1662 (41.82)	0.133
Drinking history (%)	8 (15.69)	898 (22.60)	0.240
SBP (mmHg)	146.35 ± 24.11	137.52 ± 22.95	**0.006**
DBP (mmHg)	83.82 ± 12.16	81.17 ± 11.83	0.111
HR (bpm)	79.27 ± 13.07	76.06 ± 12.13	0.060
Scr (μmol/L)	81.94 ± 12.53	76.20 ± 22.94	0.078
eGFR	79.61 ± 13.27	87.88 ± 14.18	**< 0.001**
FPG (mmol/L)	6.17 ± 1.51	5.90 ± 1.59	0.238
TC (mmol/L)	5.09 ± 1.09	5.11 ± 1.06	0.887
TG (mmol/L)	1.69 ± 0.93	1.77 ± 1.67	0.721
HDL‐C (mmol/L)	1.23 ± 0.26	1.32 ± 0.30	**0.035**
LDL‐C (mmol/L)	2.78 ± 0.75	2.67 ± 0.66	0.242
HbA1C	5.66 ± 1.03	5.36 ± 0.97	**0.034**
WBC count (10^9^/L)	6.70 ± 1.56	6.28 ± 1.87	0.112
Lymphocyte count (10^9^/L)	2.12 ± 0.58	1.94 ± 0.60	**0.039**
HGB	137.86 ± 13.59	135.99 ± 15.89	0.409
PLT (10^9^/L)	194.12 ± 49.58	193.97 ± 48.33	0.983
Hypertension (%)	17 (33.33)	791 (19.90)	**0.017**
Diabetes (%)	8 (15.69)	204 (5.13)	**0.005**
Waist circumference	86.06 ± 9.83	83.03 ± 9.76	**0.028**
Hip circumference	95.09 ± 6.64	93.17 ± 6.76	**0.045**
WHR	0.90 ± 0.06	0.89 ± 0.66	**0.013**
valvular disease history	30 (58.82)	1644 (41.37)	**0.01**
AF history	8 (15.69)	51 (1.28)	**< 0.001**

*Note:* The bold values highlight the statistically significant differences (*p* < 0.05) between the Heart Failure group and the Non‐Heart Failure group.

Abbreviations: AF, atrial fibrillation; BMI, body mass index; CNY, Chinese currency (1 CNY = 0.1572 USD); Education level; DBP, diastolic blood pressure; eGFR, estimated glomerular filtration rate; FPG, fasting plasma glucose; HbA1C, hemoglobin A1C; HDL‐C, high‐density lipoprotein cholesterol; HGB, hemoglobin; HR, heart rate; PLT, platelet count; SBP, systolic blood pressure; Scr, serum creatinine; LDL‐C, low‐density lipoprotein cholesterol; TC, total cholesterol; TG, triglycerides; WBC count, white blood cell count; WHR, waist‐to‐hip ratio.

^a^ Comparisons of category variables between groups were tested by *χ* ^2^ test or rank‐sum test (ordinal category variables) and comparisons for continuous variables between groups were tested by Student's *t* or Mann–Whitney test. Data are expressed as mean ± standard deviation or median (interquartile range) and numbers (percentage) as appropriate.

### 3.4 Evaluation of Baseline LAD on HF by Cox Regression Models

Cox regression model demonstrated the association between HF and LAD (HR = 5.721, 95% CI 3.768–8.687, *p* < 0.001) (Table 3). After adjusting for covariates including age, weight, eGFR, HDL‐C, lymphocyte count, SBP, each standard deviation increment in LAD was associated with an additional 4.69‐fold risk of ischemic stroke in the subjects. In Model 2, with further adjustments for FPG, HbA1C, waist circumference, hip circumference, valvular disease history, and MI history, each standard deviation increment in LAD remained associated with a 4.336‐fold increased risk of ischemic stroke in the subjects (*p* < 0.001).

**Table 3:** Evaluation of baseline left atrial diameter on heart failure by Cox regression models.

	Crude			Model 1			Model 2		
Variables	HR	95% CI	*p* value	HR	95% CI	*p* value	HR	95% CI	*p* value
LAD	5.721	3.768–8.687	**< 0.001**	**4.69**	2.882–7.626	**< 0.001**	4.336	2.564–7.335	**< 0.001**
Age				1.02	0.985–1.057	0.266	1.023	0.981–1.066	0.283
Weight				1.009	0.982–1.036	0.507	1.023	0.975–1.074	0.345
eGFR				0.977	0.956–0.998	0.029	0.985	0.964–1.006	0.165
HDL‐C				0.509	0.159–1.632	0.256	0.552	0.168–1.812	0.327
Lymphocyte count				1.415	1.010–1.983	0.043	1.500	1.076–2.091	0.017
Systolic blood pressure				1.002	0.991–1.015	0.683	1.003	0.991–1.015	0.61
FPG							0.943	0.737–1.206	0.638
HbA1C							1.199	0.795–1.808	0.388
Waist circumference							0.957	0.903–1.014	0.137
Hip circumference							1.041	0.983–1.102	0.171
Valvular disease history							0.159	0.067–0.374	＜0.001
AF history							0.936	0.454–1.926	0.857

*Note:* Crude: no adjustments; Model 1: adjusted for LAD, age, weight, eGFR, HDL‐C, lymphocyte count, systolic blood pressure; Model 2: adjusted for all the factors in Model 1 and FPG, HbA1C, waist circumference, hip circumference, valvular disease history, MI history. The bold values highlight the statistically significant differences (*p* < 0.05) between the Heart Failure group and the Non‐Heart Failure group.

Abbreviations: A1C; AF, atrial fibrillation; eGFR, estimated glomerular filtration rate; FPG, fasting plasma glucose; HbA1C, glycated hemoglobin; LAD, left atrial diameter; OR, odds ratio; 95% CI: 95% confidence interval.

### 3.5 Subgroup Analysis on Impact of Left Atrium Diameter on the Prevalence of HF

We performed a stratified analysis utilizing subgroups defined by age, sex, BMI, FPG, and SBP, all of which showed significant effects on HF, in order to evaluate the robustness of the Cox regression models. All analyses were adjusted for age, sex, race, family income, drinking and smoking status, serum creatinine, SBP, FPG, and BMI, with the exception of the stratified covariate. Higher LAD was consistently linked to an elevated risk of HF across all subgroups, according to Table 4, with no significant interactions found (all *p* for interaction > 0.05).

**Table 4:** Subgroup analysis on the impact of left atrium diameter on the prevalence of heart failure.

Variables	HR	Lower limit of 95% CI	Upper limit of 95% CI	*p*	*p* for interaction
Age					**< 0.001**
< 55	12.257	2.611	57.55	**0.001**	
≥ 55	5.290	2.968	9.428	< 0.001	
SBP					**<** **0.001**
< 140	12.356	4.137	36.926	< 0.001	
≥ 140	3.461	1.595	7.507	0.002	
FPG					**0.009**
< 7.0	4.819	2.577	9.012	< 0.001	
≥ 7.0	5.349	3.006	9.517	< 0.001	
Body mass index					**< 0.001**
< 28	5.191	2.842	9.482	< 0.001	
≥ 28	9.153	1.750	47.888	0.009	

*Note:* The bold values highlight the statistically significant differences (*p* < 0.05) between the Heart Failure group and the Non‐Heart Failure group.

Abbreviations: FPG, fasting plasma glucose; SBP, systolic blood pressure.

### 3.6 Lowess Model of the Correlation Between LAD and HF

Additionally, in order to explore the dose–response relationship between LAD and the risk of HF, we employed the Lowess model with a spline smoothing function fitting with full adjustment of all covariates (Figure 1B) The resultant curve displayed a linear correlation between normalized LAD and HF risk. This result was consistent with the observed increasing trend across the quartiles in the Cox regression model.

## 4 Discussion

We investigated the association between LAD and HF in a sizable inpatient sample of middle‐aged and older persons for the first time. Our study's main conclusions are twofold. Even after controlling for conventional HF risk variables and other relevant confounders, we found a significant correlation in Cox regression analysis between high LAD levels with the incidence of HF (HR = 5.721, 95% CI 3.768–8.687, *p* < 0.001). This implies that with each unit increase in LAD, the risk of HF occurrence increases by a factor of 5.721. Moreover, the correlation persisted in the age, sex, FPG, and BMI stratification analyses, suggesting that a wide range of patients can benefit from LAD's ability to stratify risk. This underscores the importance of targeted preventive programs aimed at mitigating risk factors associated with LAD. Such initiatives hold promise for reducing the prevalence of HF through both primary and secondary prevention efforts. For the purpose of therapeutically identifying patients who are more likely to get HF, LAD may be a valuable marker. Moreover, the LAD offers a way to improve HF prevention efforts by enhancing predictive risk assessment beyond conventional clinical risk variables. This suggests the potential for LAD measurement to refine risk estimation and optimize HF prevention efforts. Furthermore, in ultrasound practice, it guides examinations and facilitates precise risk assessment and treatment planning. Incorporating the LAD threshold into predictive models enhances their accuracy, effectively identifying high‐risk HF populations. This advancement supports tailored interventions, such as pharmacotherapy and lifestyle modifications, to mitigate HF risk and improve outcomes.

The concept of LA remodeling has evolved over the years, with an increased understanding of the LA's role in the pathophysiology of HF [20, 21, 22]. Indeed, changes in the structure and function of the left atrial chamber may serve as indicators of HF severity or contribute to the development of cardiac dysfunction. Atrial fibrillation, a common symptom of LA disease, notably increases morbidity in HF patients. Furthermore, aging and endurance activities are associated with LA enlargement even in the absence of LA disease [23, 24]. In patients with HF, impaired atrial contractile reserve and left ventricular enlargement are independent predictors of death, cardiovascular morbidity, onset or occurrence of atrial fibrillation, and the degree of functional impairment [25, 26, 27, 28].

Understanding cardiac physiology and pathology may help to explain why the LAD has such great promise as a HF risk predictor. By acting as a reservoir for pulmonary venous return during ventricular systole, a conduit for pulmonary venous return during early ventricular diastole, and a booster pump that increases ventricular filling during late ventricular diastole, the LA primarily regulates left ventricular filling and cardiovascular performance. The left atrial chamber is exposed to the diastolic pressure of the left ventricle during diastole, with the exception of the isovolumic relaxation phase, due to the open mitral valve. Pathologic LA remodeling is driven by the LA pressure and volume overload brought on by either atrial or functional mitral regurgitation. Therefore, the atrium is considered the initial site where the impact of HF begins to manifest in the heart's development.

Literature suggests that myocardial remodeling may occur in the LA independent of the left ventricle [29, 30, 31]. In addition to the mechanical changes detailed above, HF is associated with a variety of neurohormonal alterations, including increased levels of norepinephrine and endothelin [32, 33, 34]. Consequently, the escalation in afterload exacerbates the advancement of LA myopathy, resulting in a greater accumulation of LA fibrosis. This reactive response of the LA to increased afterload also provides theoretical support for predicting HF from LA conditions [33, 34]. Patients who are prone to developing HF often exhibit overactivation of the renin–angiotensin–aldosterone system. When subjected to angiotensin II treatment, the LA demonstrated elevated levels of profibrotic transcription factors and metalloproteinases compared to the RA. The LA displays increased expression of genes associated with higher intracellular Ca^2+^ levels and fibroblast activity, including Pitx2c, profibrotic protease chymase, and calcitonin gene‐related peptide [35, 36], while also showing lower levels of inhibitory messengers of profibrotic cytokine signaling, such as Smad 6 [37, 38, 39, 40]. The LA's predisposition to readily respond to substances that promote remodeling provides the potential for LAD to serve as a predictive factor for HF.

LA remodeling is often accompanied by metabolic and oxidative stress, as evidenced by literature references [41, 42, 43]. In the coexistence of HF and atrial fibrillation, the energy metabolism disturbance in the atria is more pronounced compared to the ventricles [44]. Energy depletion has been linked to aberrant mitochondrial functional and ultrastructural remodeling as well as decreased mitochondrial oxidative phosphorylation complexes. Atrioventricular effective refractory period lengthening, fibrosis, electrical instability with collapsed membrane potential, changed action potential morphology, and impaired ionic homeostasis can all result from defective mitochondrial function, pathways, mtDNA, and ultrastructure [41, 45, 46]. This could explain why people who already have atrial fibrillation are more likely to develop HF.

### 4.1 Strengths and Limitations

While widely utilized, traditional models, such as the Framingham HF Risk Score and Health ABC Heart Failure Score, notably overlook echocardiography indicators. Echocardiography, a standard tool for immediate cardiac function assessment, holds untapped potential in forecasting HF risk. We have identified LAD as a pivotal indicator that significantly enhances HF risk prediction. This revelation not only refines risk estimation but also optimizes HF prevention strategies. Incorporating the LAD threshold into predictive models markedly improves accuracy, enabling the precise identification of high‐risk HF populations. Consequently, tailored interventions, including pharmacotherapy and lifestyle modifications, can be initiated promptly to mitigate HF risk and enhance patient outcomes. Moreover, the integration of the LAD into ultrasound practice facilitates targeted examinations and enhances risk assessment and treatment planning precision. Overall, our study underscores how the critical LAD threshold significantly augments HF prediction accuracy, enabling personalized care and improving patient prognosis across clinical and ultrasound applications.

Several limitations are noteworthy in our study. First, the majority of our HF patients exhibited severely depressed left ventricular systolic function, which precluded exploration of the relationship between HF types and LAD. This limitation reflects the diagnostic criteria utilized for HF during the study period and impedes the extrapolation of our results to patients with preserved systolic function. Second, the majority of patients in our study are from Eastern China, which may limit the generalizability of our findings to other ethnic groups residing in different regions of the country. Additionally, potential residual confounding by unreported or unknown risk factors could bias our results, as is common in observational studies. However, our findings remained robust even after adjusting for several significant risk factors. Despite these limitations, our study has several strengths, including a substantial patient cohort, a long‐term follow‐up design, and the use of standardized and well‐documented data collection tools, all contributing to enhanced statistical power.

## Conflicts of Interest

The authors declare no conflicts of interest.

## Data Availability

The data that support the findings of this study are available from the corresponding author upon reasonable request.
